# Detection of Pb(II): Au Nanoparticle Incorporated CuBTC MOFs

**DOI:** 10.3389/fchem.2020.00803

**Published:** 2020-10-15

**Authors:** Gajanan A. Bodkhe, Bhavna S. Hedau, Megha A. Deshmukh, Harshada K. Patil, Sumedh M. Shirsat, Devdatta M. Phase, Krishan K. Pandey, Mahendra D. Shirsat

**Affiliations:** ^1^RUSA Center for Advanced Sensor Technology, Department of Physics, Dr. Babasaheb Ambedkar Marathwada University, Aurangabad, India; ^2^Department of Electronics and Telecommunication Engineering, Jawaharlal Nehru Engineering College, Aurangabad, India; ^3^UGC-DAE Consortium for Scientific Research, University Campus, Indore, India; ^4^High Pressure and Synchrotron Radiation Physics Division, Bhabha Atomic Research Center, Mumbai, India

**Keywords:** differential pulse voltammetry (DPV), electrochemical sensor, gold nanoparticles, metal organic frameworks, CuBTC MOF

## Abstract

In the present investigation, copper benzene tricarboxylate metal organic frameworks (CuBTC MOF) and Au nanoparticle incorporated CuBTC MOF (Au@CuBTC) were synthesized by the conventional solvothermal method in a round bottom flask at 105°C and kept in an oil bath. The synthesized CuBTC MOF and Au@CuBTC MOFs were characterized by structure using X-ray diffraction (XRD) spectroscopic methods including Fourier Transform Infrared spectroscopy, Raman Spectroscopy, X-ray Photoelectron Spectroscopy (XPS), and Energy dispersive spectroscopy (EDS). We also characterized them using morphological techniques such as Field emission scanning electron microscopy (FE-SEM), and electrochemical approaches that included cyclic voltammetry (CV) and electrochemical impedance spectroscopy (EIS). We examined thermal stability by thermogravimetric analysis (TG/DTA) and N_2_ adsorption—desorption isotherm by Brunauer-Emmett-Teller (BET) surface area method. Both materials were tested for the detection of lead (II) ions in aqueous media. Au nanoparticle incorporated CuBTC MOF showed great affinity and selectivity toward Pb^2+^ ions and achieved a lower detection limit (LOD) of 1 nM/L by differential pulse voltammetry (DPV) technique, which is far below than MCL for Pb^2+^ ions (0.03 μM/L) suggested by the United States (U.S.) Environmental Protection Agency (EPA) drinking water regulations.

## Introduction

Heavy metal ions are a major water pollutant caused by sewage from chemical industries and various other sources (Singh et al., 2011; Maleki et al., 2019). Some of the more toxic heavy metal ions include Pb(II), Cu(II), Hg(II), Co(II), etc. (Meena et al., 2005). The accumulation of heavy metallic ions results in severe health issues, damage to the ecosystem (Wu et al., 2010), and significant damage to the environment (Fu and Wang, 2011). It is well-known that Pb(II) is toxic for human and aquatic health (Deshmukh et al., 2018b), causing health issues which include memory damage, muscle fatigue, neurotic diseases, damage to the liver and kidneys, reduction in hemoglobin formation, infertility, and abnormalities in pregnancy, amongst other concerning side effects (Meena et al., 2005). There is an urgent need to detect and remove these pollutants from water, by introducing highly sensitive and selective sensing materials. Various technologies have been introduced for highly sensitive heavy metal ion detection, namely liquid chromatography (Ali and Aboul-Enein, 2002), solid-phase extraction coupled with atomic absorption spectroscopy (Faraji et al., 2010), and atomic emission spectroscopy. However, these methods are very expensive, time-consuming, and require experts to operate the instruments (Quang and Kim, 2010).

Researchers are also dedicating efforts to developing heavy metal ions sensors in other modalities, such as the fluorescent (Neupane et al., 2011), Field Effect Transistors (FETs) (Cobben et al., 1992), Surface-Enhanced Raman Scattering (SERS) (Tan et al., 2012), Surface Plasmon Resonance (SPR) (Forzani et al., 2005), colorimetric (Hung et al., 2010), and electrochemical sensors (Deshmukh et al., 2018c,d). Most of these techniques are challenging due to the high cost of the laboratory facilities that they require. Modalities such as electrochemical sensors are, on the other hand, an ideal and prominent option due to advantages such as the fact that they require simple and cost-effective instruments and have faster analytical responses (Kimmel et al., 2011). Researchers have explored various materials for the detection of heavy metal ions in aqueous media in electrochemical modality, for example considering organic conducting polymers (OCPs) (Deshmukh et al., 2018e), carbon nanotubes (CNTs) (Deshmukh et al., 2018e; Maleki, 2018), metal oxides (Hwang et al., 2008), graphene (Dai et al., 2016), bioreceptors (Cui et al., 2015), metal nanoparticles (Kim et al., 2001), and metal organic frameworks (MOFs) (Roushani et al., 2016), among others.

Recently, researchers have had an interest in an efficient material called Metal Organic Frameworks (MOFs) due to versatile physical and chemical properties, high porosity, ultrahigh surface area, high electrical transfer rate, high crystallinity, evenly element doping, and abundant metal sites (Bodkhe et al., 2019), among other benefits. MOFs are generally composed of a central metal ion and organic linker resulting in crystalline and porous materials. These properties make them useful in versatile applications as a catalyst (Lee et al., 2009), potential biomedical applications (Wuttke et al., 2017), sensors (Rauf et al., 2020), gas storage (Chen et al., 2020), and separation (Lin et al., 2020).

However, the sensing properties of MOFs can be enhanced by combining them with other efficient materials (Liu et al., 2018). Nanocomposites (Maleki et al., 2016) are a combination of various materials that overcome or improve deficiencies in the parent material (Maleki et al., 2018), which makes them advantageous when used in sensors. The episodic structure of MOFs makes them an attractive candidate for the encapsulation of metal nanoparticles (such as Ag, Pt, Cu, and Au, etc.) due to increased control of the regular distribution of nanoparticles and enhancement of the stability of NPs (Butova et al., 2018). The porous structure of MOFs make NPs accessible to the environment, meaning they are good candidates for use in sensors. Among all nanoparticles, gold (Au) nanoparticles have shown better results when incorporated in MOFs, showing carbon monoxide oxidation (Jiang et al., 2009), alcohols oxidation (Ishida et al., 2008), hydrazine electrocatalytic oxidation (Han et al., 2015), and a reduction of 4-hydroxynitrobenzene (Ke et al., 2015).

Inspired by these reports, this study exploited various properties of copper benzene tricarboxylate (CuBTC) MOF to synthesize the Au nanoparticles incorporated CuBTC (henceforth referred to as Au@CuBTC). CuBTC MOF contains Cu(II) central metal ion and benzene tricarboxylic acid as an organic linker. CuBTC MOF is the most explored MOF. The CuBTC MOF shows good electrochemical performance and electro catalytic activity which makes them suitable for electrochemical sensors using hydrazine (Meng et al., 2019), nitrite (Saraf et al., 2016), hydroquinone, catechol (Zhou et al., 2015), ammonia (Travlou et al., 2015), and H_2_O_2_ (Zhang et al., 2013; Golsheikh et al., 2020). This study observed that pure CuBTC MOF does not show any electrochrochemical response to Pb(II) ions at 0.01 mM/L concentration. However, Au@CuBTC MOF shows an electrochemical response up to 1 nM/L concentration, which is far below the MCL level of Pb(II) ions. The porous structure of CuBTC provided maximum surface area and Au NPs enhanced electrocatalytic activity, which makes the material a highly sensitive and selective electrochemical sensor for Pb(II) ions.

## Experimental

### Materials and Chemicals

All the reagents and chemicals were analytical grade and used without purification. Benzene-1,3,5-tricarboxylic acid (BTC), Copper nitrate trihydrate (Cu(NO_3_)_2_,3H_2_O), and N, N dimethyl formamide (DMF) purchased from Sigma Aldrich. Chloroauric acid, Hydrazine hydrate, hydrochloric acid, sodium acetate, acetic acid, sodium hydroxide, hydrochloric acid, and ethanol purchased from Molychem, India.

### Synthesis of CuBTC MOF

The CuBTC MOF was synthesized as per the reported method (Marx et al., 2011). 2.252 gm of Copper nitrate trihydrate [Cu(NO_3_)_2_, 3H_2_O] was mixed with 50 ml of deionized water and benzene-1,3,5-tricarboxylic acid (BTC) (0.982 gm) in 50 ml of DMF, prepared separately and mixed with constant stirring at room temperature. The precursor solution was transferred into a 250 ml round-bottom flask and kept in an oil bath at 105°C for 4 h. After completion of the reaction, CuBTC MOF crystals were observed at the bottom of the round-bottom flask. After cooling at room temperature, CuBTC MOF crystals were washed with deionized water and ethanol three times each to remove unreacted reactants. The filtered CuBTC MOF was kept for activation at 120°C for 12 h to remove coordinated solvent molecules from the pores (Bodkhe et al., 2019). After activation, the color of CuBTC changed from faint blue to dark blue. The activated CuBTC MOF was stored in air tight sample containers for further use.

### Synthesis of Au@CuBTC MOF

Gold (Au) nanoparticle precursor solution was prepared separately, as reported in other studies (Yadav et al., 2018). Chloroauric acid (HAuCl_4_) (0.71 mM) was used as the gold (Au) source, mixed with 0.1 M hydrochloric acid (HCl) in D. I. water, with 0.71 mM hydrazine hydrate then added slowly. This solution was stirred for 30 min and sonicated for 5 min. The synthesis procedure for Au@CuBTC MOF is the same as CuBTC MOFs. In the precursor solution of CuBTC, the Au NPs precursor solution was added slowly then stirred for 30 min and sonicated for 5 min. This solution was transferred into a 250 ml round bottom flask and kept in an oil bath for 4 h at 105°C. Au@CuBTC MOF was cooled at room temperature and washed with deionized water and ethanol three times each. The filtered Au@CuBTC MOF activated at 120°C for 12 h and stored for further use.

### Preparation of CuBTC and Au@CuBTC MOF Electrode

Glassy carbon electrode (GCE) was used as an electrode for all electrochemical experiments. The GCEs were carefully polished with alumina powder slurries of 1, 0.3, and 0.05 μ, respectively, to get a mirror like surface. Then the GCE was ultrasonicated and washed with acetone and D.I. water sequentially. The preconditioning of bare GCE was performed in 0.5 M of H_2_SO_4_ solution using the cyclic voltammetry (CV) technique in the potential window of −0.35 to 1.5 V at a scan rate of 100 mV/s until stable voltammogram was observed.

The pure CuBTC and Au@CuBTC MOF were properly mixed in diluted nafion in acetone. The prepared suspension of pure CuBTC and Au@CuBTC MOF were drop cast on GCE electrodes and dried at room temperature, then used for electrochemical experiments.

### Electrochemical Detection of Lead (Pb^2+^) Ions

The Pb^2+^ ions solution was prepared in acetate buffer at pH 5.0 using Pb(NO_3_)_2_ (Pb^2+^ ions source), Pb(NO_3_)_2_ mixed properly, with constant stirring for 30 min. The Pb^2+^ ions solution was prepared at a higher concentration and used as a stock solution, a lower concentration was prepared by diluting it with the addition of a buffer solution. Electrochemical detection of Pb^2+^ ions was carried out on CHI 660C electrochemical workstation by the differential pulse voltammetry technique. The GCE modified electrodes of pure CuBTC and Au@CuBTC were used as the working electrode, platinum served as the counter electrode, while Ag/AgCl were used as a reference. Initially, the accumulation of Pb^2+^ ions on GCE modified electrodes was carried out by dipping the modified electrode for 5 min at constant pH of 5.0, then DPV in another buffer (pH = 5.0) was carried out in the potential from 0 to (−1) volts.

### Apparatus and Instruments

Powder X-ray diffraction (XRD) measurements were performed on a Bruker D8 Advance Diffractometer with Cu kα1 radiation (λ = 1.5406). The Fourier Transform Infrared (FTIR) spectrum was recorded on Bruker Alpha FTIR in ATR mode with the ZnSe window in the range of 500 to 4,000 cm^−1^. Nitrogen adsorption-desorption isotherm measurements were carried out on Autosorb iQ Station 1 instrument at 77 K and all samples were degassed at 150°C in a helium environment. The X-Ray Photoelectron studies were carried out on the XPS facility available at Raja Ramanna Center for Advanced Technology (RRCAT), Indore, India.

Thermogravimetric measurements were carried out on Shimadzu to take the DTG-60H model from room temperature to 1,000°C, with a scan rate of 10°C/min in the nitrogen environment. The Raman spectrum of all the samples was recorded on a Renishaw inVia reflex Raman microscope in the spectral range of 90 to 2,000 cm^−1^ at 532 nm laser excitation wavelength. Cyclic Voltammetry (CV) and Electrochemical Impedance Spectroscopy (EIS) measurements were carried out on a CHI 660C electrochemical workstation with platinum (Pt) foil, Ag/AgCl as counter and reference electrodes, respectively. The CV was recorded in the potential range of −0.8–0.5 V in 10 mM [Fe(CN)6]^3−/4−^ solution containing 0.1 M KCl solution at a scan rate of 100 mV/S. EIS measurements were taken in the frequency range of 0.01–0.1 MHz in the 0.1 M KCl solution. Electrochemical sensing experiments were performed in acetate buffer at 5.0 pH by differential pulse voltammetry (DPV) technique on CHI 660C electrochemical workstation having Ag/AgCl and Pt foil as reference and counter electrodes respectively.

## Results and Discussion

### Materials Characterization

#### Fourier Transform Infrared Spectroscopy Analysis

**Figure 1:**
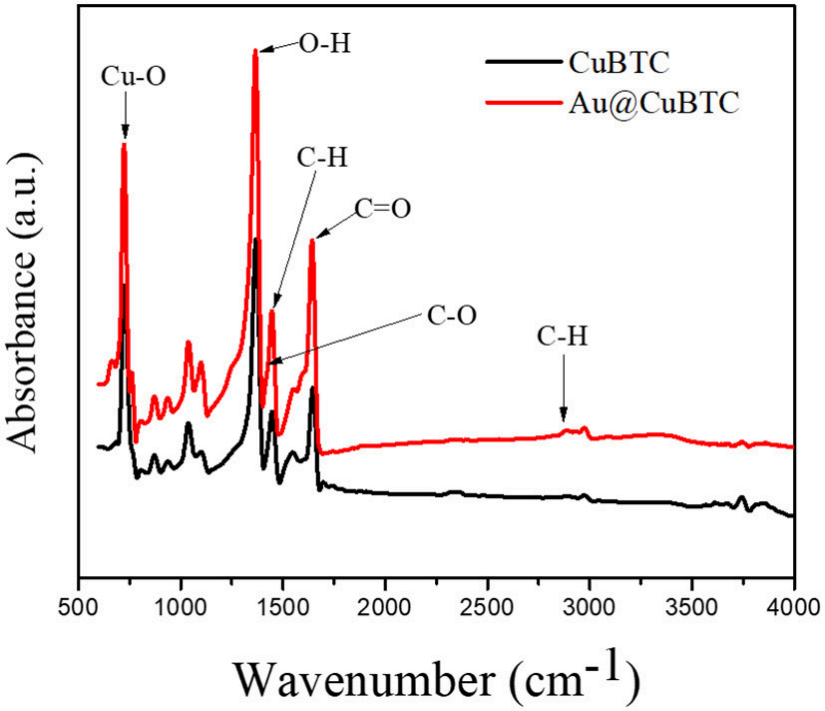
FTIR Spectra of as-synthesized CuBTC MOF and Au@CuBTC MOF.

As-synthesized CuBTC MOF and Au@CuBTC MOF were characterized by FTIR spectroscopy as shown in Figure 1. In CuBTC MOF, the presence of asymmetric C-H stretching vibration and asymmetric stretching of the carboxylic groups confirmed by bands present at 2,890 cm^−1^ and 1,446 cm^−1^ (Dastan et al., 2016). The absorption band at 730 cm^−1^ attributed to Cu-O stretching vibration. The absorption peaks at 1,640 cm^−1^ attributed to the stretching of V_C = O_ while the band at 1,446 cm^−1^ were attributed to the stretching of V_C−O._ The band at 1,373 cm^−1^ indicated that there was a bending of O-H functional groups (Lin et al., 2014) and confirms the CuBTC MOF. The FTIR spectra of Au nanoparticle incorporated CuBTC does not show any difference in peak position in FTIR spectra, but there was an increase in absorption of the IR spectrum in Au nanoparticle incorporated CuBTC MOF. This confirms that no vibrational bond formed between CuBTC MOF and Au nanoparticles.

#### Raman Spectroscopy Analysis

**Figure 2:**
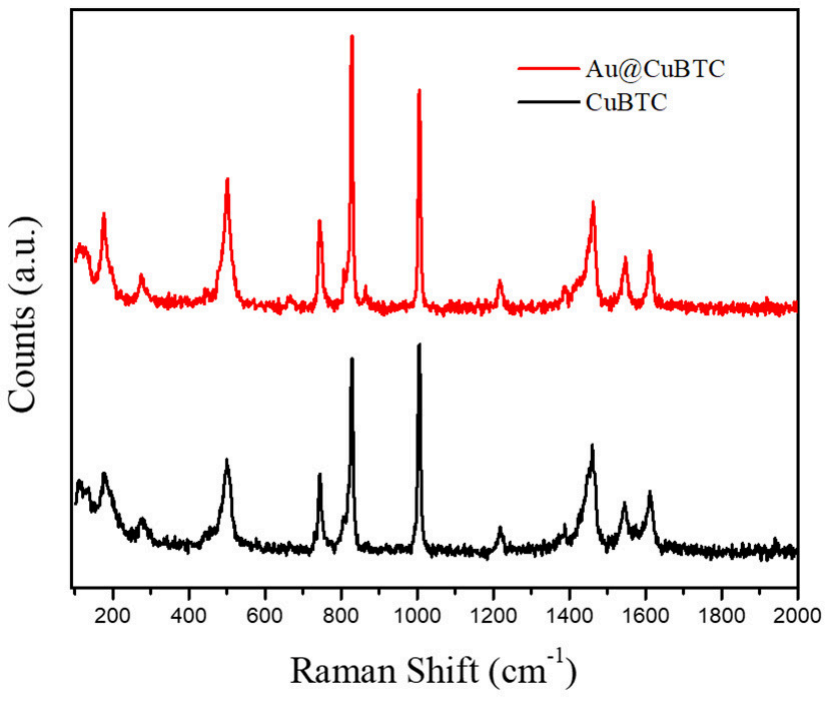
The Raman Spectra of as-synthesized CuBTC MOF and Au@CuBTC MOF.

The Raman spectra of CuBTC MOF and Au nanoparticle incorporated CuBTC MOF are shown in Figure 2. The Raman spectra of CuBTC MOF shows that the Raman shift at 500 cm^−1^ corresponds to the presence of Cu(II) species, with peaks at 1,006 cm^−1^ and 1,606 cm^−1^ attributed to the ν(C=C) modes of the benzene ring. The vibrational peak at 1,457 cm^−1^ corresponds to ν_s_(COO^−^). This confirms the formation of CuBTC MOF (Deshmukh et al., 2018b). Au@CuBTC MOF does not show any change in the Raman spectra Au nanoparticles, does not affect the chemical bonding in CuBTC MOF, and that the Au nanoparticles are not Raman active (Worrall et al., 2016).

#### Structural Analysis

**Figure 3:**
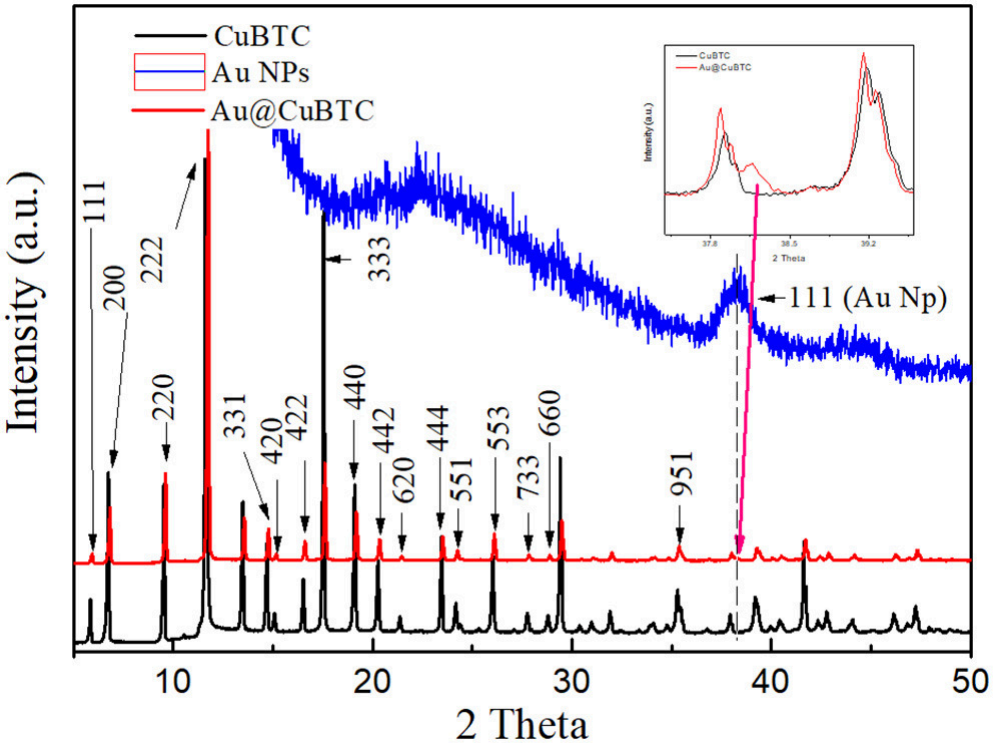
XRD of as-synthesized CuBTC MOF and Au@CuBTC MOF.

The structural analysis of as synthesized CuBTC and Au@CuBTC MOF carried out by X-ray diffraction is shown in Figure 3. All the diffraction peaks were compared and matched with the ICDD database no. PDF 00-065-1028 and reported literature (Wang et al., 2014). Both the patterns of as synthesized CuBTC and Au@CuBTC shows the same diffraction pattern and peak positions except one very small extra peak, which appeared at 2θ = 38.2°C. This confirms the presence of Au nanoparticles in the compound, while peaks in both materials represent the crystal structure of CuBTC MOF (cubic) at the 2θ angle with respective d_hkl_ planes showing 5.8° (111), 6.7° (200), 9.5° (220), 11.6° (222), 14.7° (331), 15° (420), 16.5° (422), 17.5° (333), 19,0° (440), 20.2° (442), 21.3° (620) 23.4° (444), 24.1° (551), 26.0° (553), 27.7° (733), 28.7° (660), and 35.2° (951). The crystalline size of both the compounds was calculated by classical Scherrer formula (Dastan et al., 2014) by considering peaks of the XRD patterns, as shown in Figure 3:

(1) $$\begin{array}{r} \text{D }=\;\frac{\text{Kλ}}{\beta \text{Cosθ}} \end{array}$$

where *D* is the average crystallite size, λ is the wavelength of the X-ray radiation (λ = 1.5404 A°), *K* is the Scherrer constant (0.89 for cubic shape), θ is the Bragg diffraction angle, and β is the full width at half-maximum height (FWHM). The crystallinity and crystallite size of as synthesized CuBTC were found to be 74.4% and 199.94 nm, respectively, while reduction of the crystallinity and crystallite size in Au nanoparticle incorporated CuBTC MOF observed as 69.9% and 108.41 nm respectively.

#### Morphological Analysis

**Figure 4:**
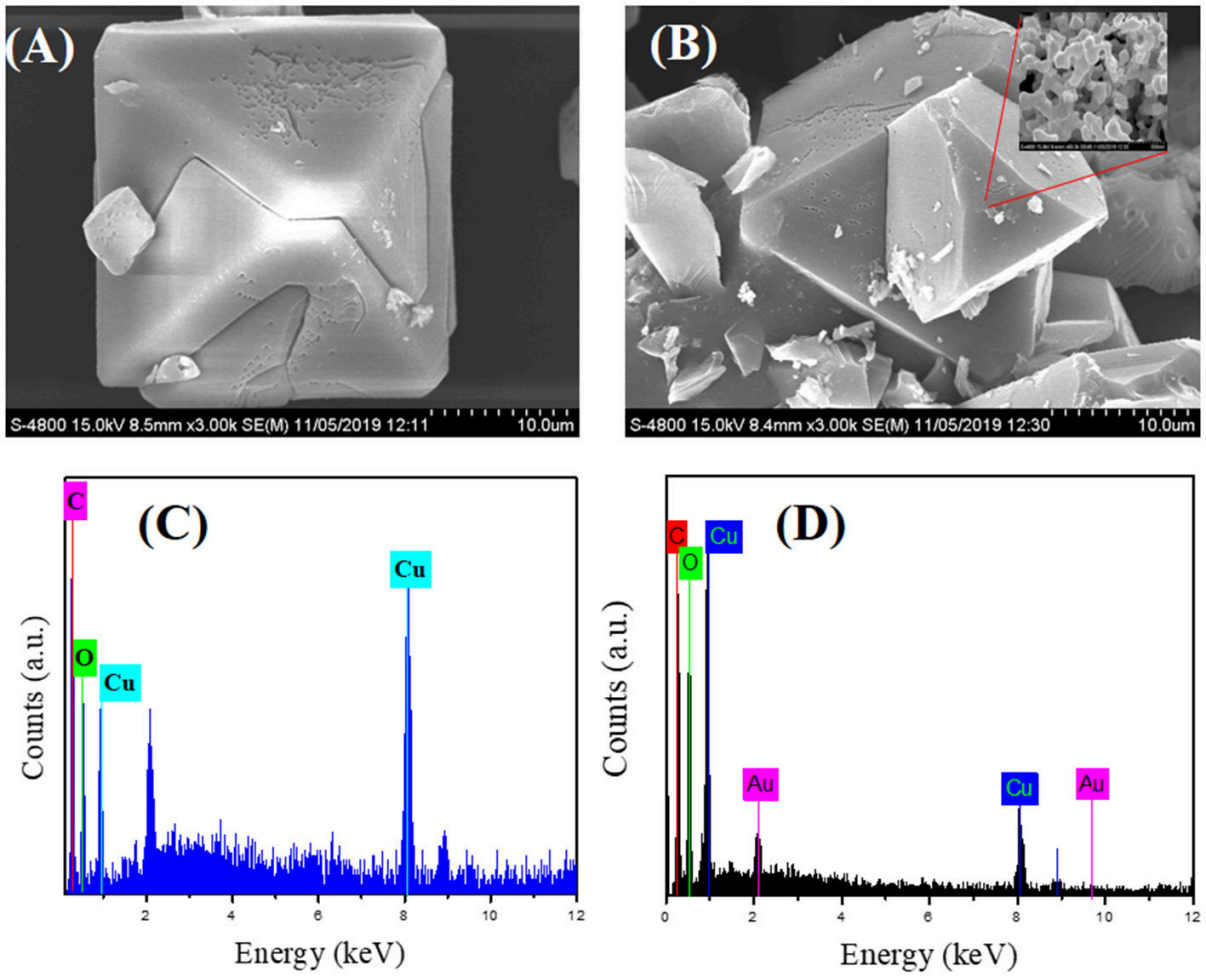
FE-SEM images of **(A)** as-synthesized CuBTC, **(B)** Au@CuBTC, and Energy-dispersive X-ray spectroscopy (EDX) analysis of **(C)** as-synthesized CuBTC, **(D)** Au@CuBTC.

The FE-SEM images show distinct changes in morphology, particle size, and the crystalline nature of as-synthesized CuBTC, Au@CuBTC, shown in Figures 4A,B. Figure 4B indicates the presence of the Au nanoparticles in the composite on the surface, but most that the Au nanoparticles are incorporated in the MOF crystal. The elemental confirmation of the compounds was carried out using Energy-dispersive X-ray spectroscopy (EDX). Inside the CuBTC MOF structure, we recorded EDX spectra (Figures 4C,D) and found the presence of the Au NPs inside the CuBTC MOF, shown in Figure 4D. We also found the elemental concentration detail of Au@CuBTC, which is shown in Table 1. The CuBTC MOF has a cubic structure with a high surface area and porous morphology that provides more active sites for Au NPs inside the CuBTC MOF for the detection of Pb (II) ions sensitively.

**Table 1:** EDS elemental analysis data of as-synthesized Au@CuBTC.

**Element**	**Atomic no.**	**Unnormalised concentration (wt%)**	**Normalized concentration (wt.%)**	**Atom concentration (%)**	**Error (wt%) (1 sigma)**
Carbon (C)	6	26.05	34.19	56.28	5.50
Oxygen (O)	8	19.53	25.63	31.67	4.09
Copper (Cu)	29	28.99	38.04	11.84	1.51
Gold (Au)	79	1.63	2.14	0.22	0.16
Sum		76.21	100.00	100.00	-

#### Thermogravimetric Analysis

**Figure 5:**
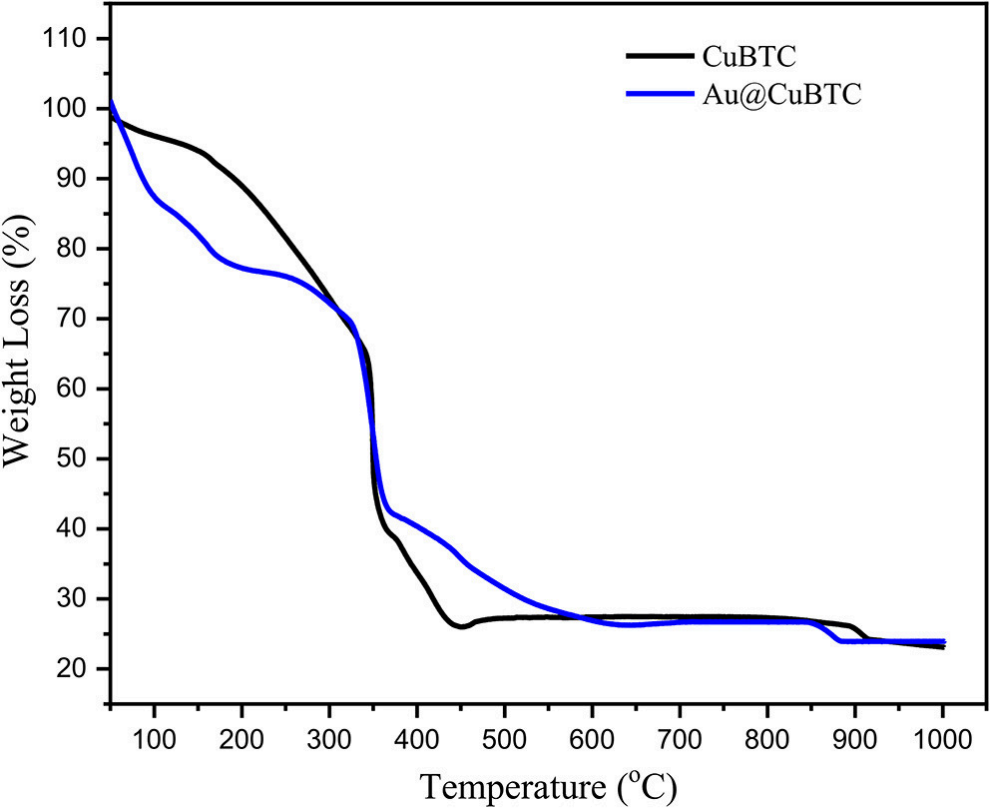
Thermogravimetric (TGA) analysis of as synthesized CuBTC, and Au@CuBTC MOF.

The thermogravimetric analysis of as-synthesized CuBTC and Au nanoparticles incorporated CuBTC is shown in Figure 5. In as-synthesized CuBTC, small weight loss (5.4%) started from 40 to 133°C due to the removal of H_2_O molecules, while a weight loss of 29.8%, from 133 to 343°C was observed, due to the dislodgement of coordinated DMF molecules from the CuBTC MOF Structure. There was an obvious weight loss above 343°C of ~49% in the CuBTC MOF network, which produced H_2_O, CO_2_, and Cu_2_O, meaning that CuBTC MOF is thermally stable up to 343°C. In Au@CuBTC, a small weight loss (15.16%) started from 40 to 122°C due to the removal of the H_2_O molecule. A weight loss of (16.9%) from 133 to 180°C was observed, which is due to the dislodgement of coordinated DMF molecules from the Au@CuBTC MOF Structure. An obvious weight loss of ~41.78% above 335°C was observed in Au@CuBTC MOF and produced H_2_O, CO_2_, and Cu_2_O. These results confirm that the thermal stability of both the materials is almost the same up to ~335°C.

#### Brunauer-Emmett-Teller (BET) Analysis

**Figure 6:**
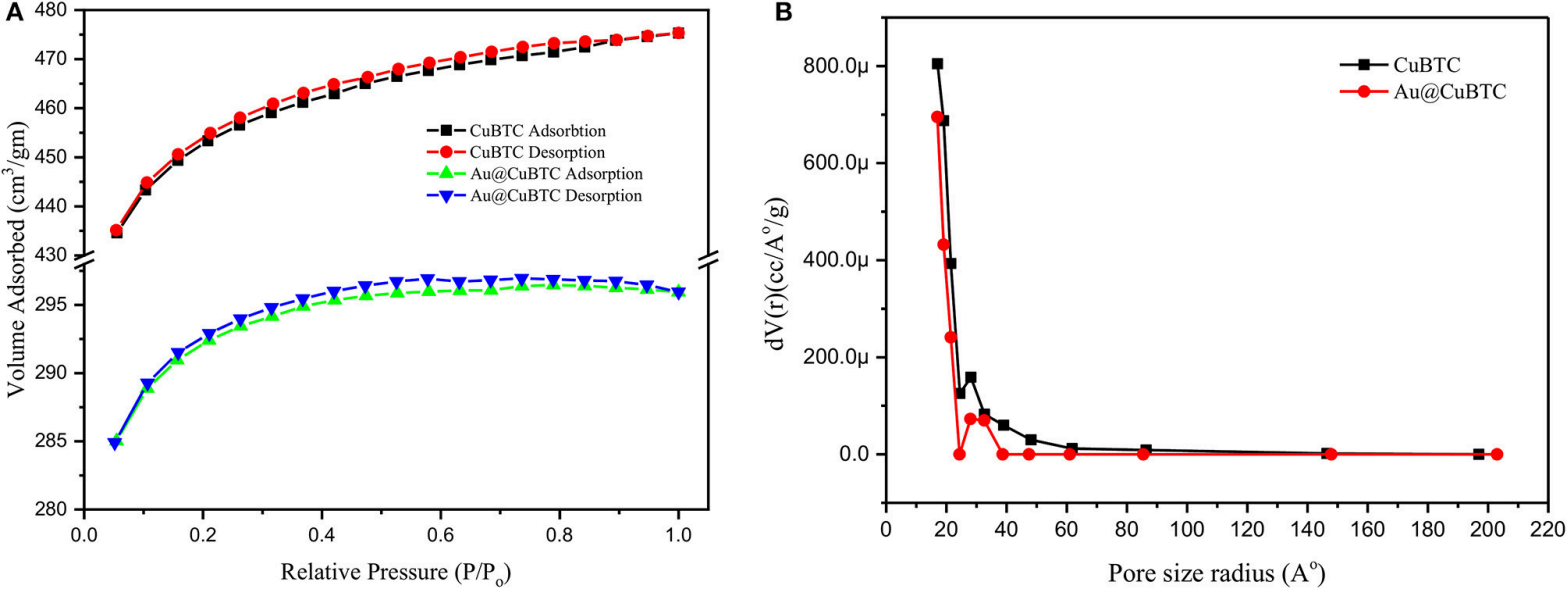
**(A)** N_2_ adsorption-desorption isotherm and **(B)** BJH Pore size distribution of as synthesized CuBTC and Au@CuBTC.

N_2_ adsorption-desorption isotherm and BJH Pore size distribution of the as synthesized CuBTC and Au@CuBTC was studied using the Brunauer-Emmett-Teller (BET) technique, shown in Figures 6A,B, respectively. Both the MOFs were degassed in the Helium environment at 150°C to remove possible contaminations inside the pores. The physical adsorption of nitrogen at a temperature of 77 K was carried out for the BET surface area and pore size measurement. Both the compounds clearly show a microporous structure, having type I adsorption-desorption isotherm (Akhtar et al., 2018). The BET surface area details are shown in Table 2. The surface area, total pore volume, and average pore radius of Au nanoparticle incorporated CuBTC MOF reduces as compared to the synthesized CuBTC MOF.

**Table 2:** BET surface area details.

**Compound**	**Surface area (m^**2**^/gm)**	**Total pore volume (cc/gm)**	**Average pore radius (nm)**
CuBTC	1374.983	0.7352	1.06942
Au@CuBTC	876.704	0.4589	1.04691

#### X-ray Photoelectron Spectroscopy (XPS) Analysis

**Figure 7:**
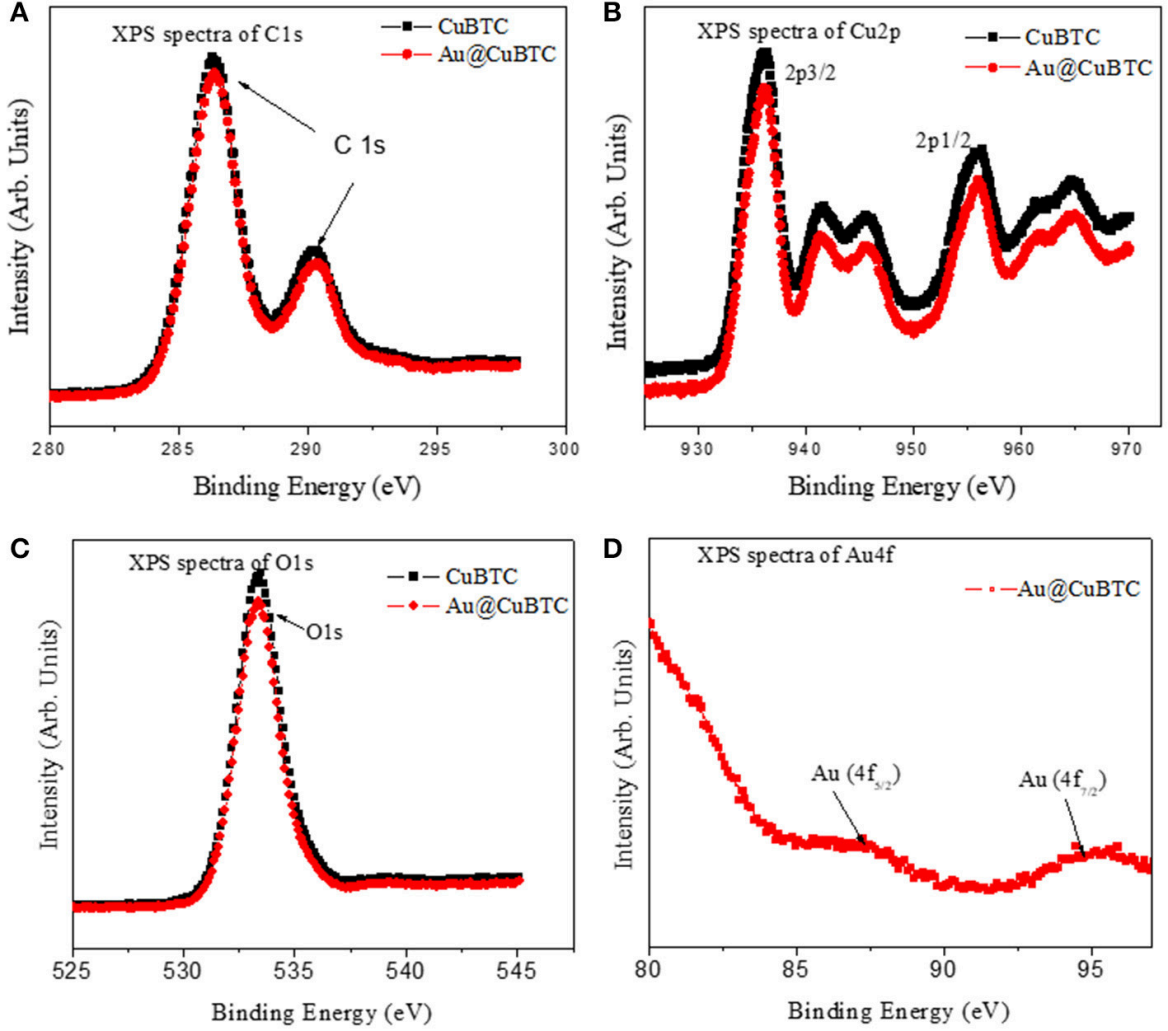
XPS spectra of **(A)** C1s **(B)** Cu2p **(C)** O1s of CuBTC and Au@CuBTC and **(D)** Au4f of Au@CuBTC.

To analyze the surface chemical state of as synthesized CuBTC and Au@CuBTC, XPS measurements were performed as shown in Figure 7. The XPS spectra of C 1s (Figure 7A) shows binding energy (BE) peaks at 286.3 and 290.2 eV of as synthesized CuBTC and Au@CuBTC, with the same BE of C 1s. This indicates no interaction between Au nanoparticle and CuBTC at the carbon site. The XPS spectra at Cu 2p (Figure 7B) of as synthesized CuBTC and Au@CuBTC shows the same spectrum having a small decrease in peak intensity of Au@CuBTC, which may be due to the presence of Au nanoparticles inside the CuBTC. The peaks present in both the compounds at 936.2 eV and 956.3 eV correspond to Cu 2p3/2 and Cu 2p1/2, with shake-up satellite bands that confirm the +2 oxidation state (Luo et al., 2016). The spectra of O 1s (Figure 7C in both the compound shows no change in the chemical states of oxygen after incorporation with Au nanoparticles in CuBTC. The peak at BE of 533.4 eV indicates the presence of carboxylic species in CuBTC. Figure 7D shows the XPS spectra of Au@CuBTC at the binding energy edge of Au 4f and confirms the presence of Au nanoparticles in CuBTC, with the peaks at 87.30 and 94.70 eV attributed to the Au 4f5/2 and Au 4f7/2 transitions (Ghodake et al., 2010).

#### Electrochemical Analysis

**Figure 8:**
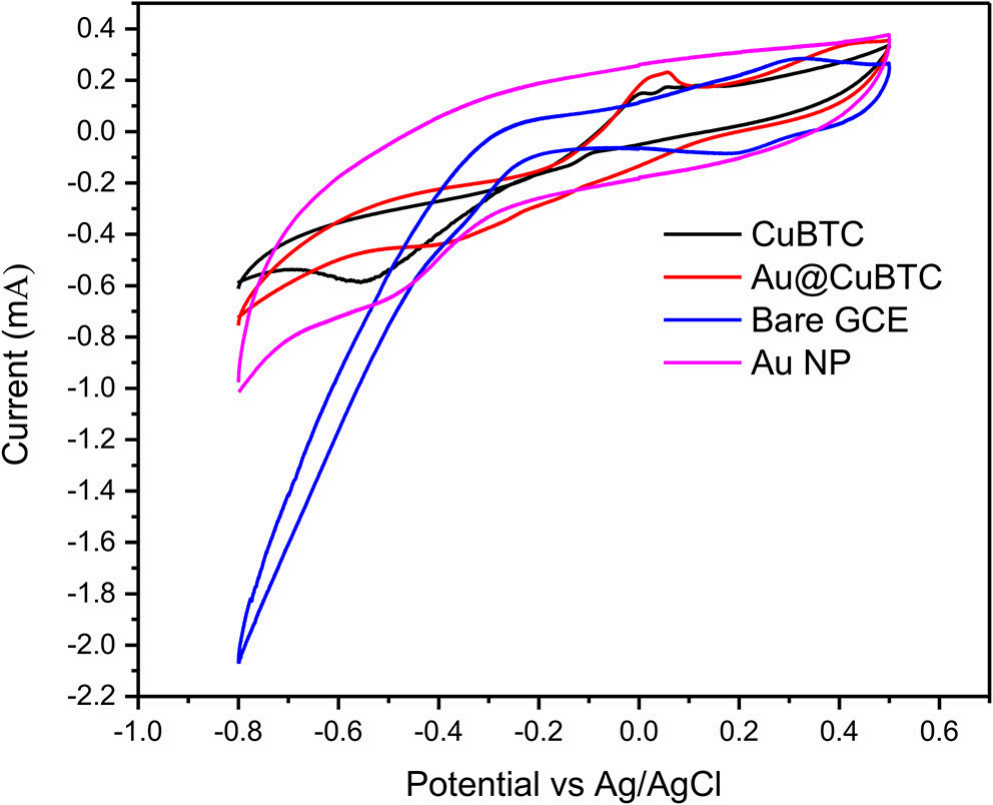
Cyclic voltammograms (CV) of bare GCE, Au NP, CuBTC MOF, and Au@CuBTC MOF.

The electrochemical activity of bare GCE, Au Np, CuBTC, and Au@CuBTC were studied by cyclic voltammetry in a 10 mM [Fe(CN)_6_]^3−/4−^ solution containing 0.1 M KCl solution at a scan rate of 100 mV/s, as shown in Figure 8. The CuBTC coated glassy carbon electrode (GCE) shows distinct oxidation and reduction peaks at 0.02 and −0.5 V versus Ag/AgCl reference electrode, indicating the reversible oxidation and reduction of Cu(II) to Cu(I) (Kumar et al., 2012). The CV curve of the Au@CuBTC coated GCE electrode shows a distinct reduction peak compared with pure CuBTC and the same oxidation peak, with an increase in oxidation current that probably an overlapping of the oxidation of Au nanoparticles over CuBTC MOF, while bare GCE and Au Np coated GCE shows higher current due to high conductivity.

**Figure 9:**
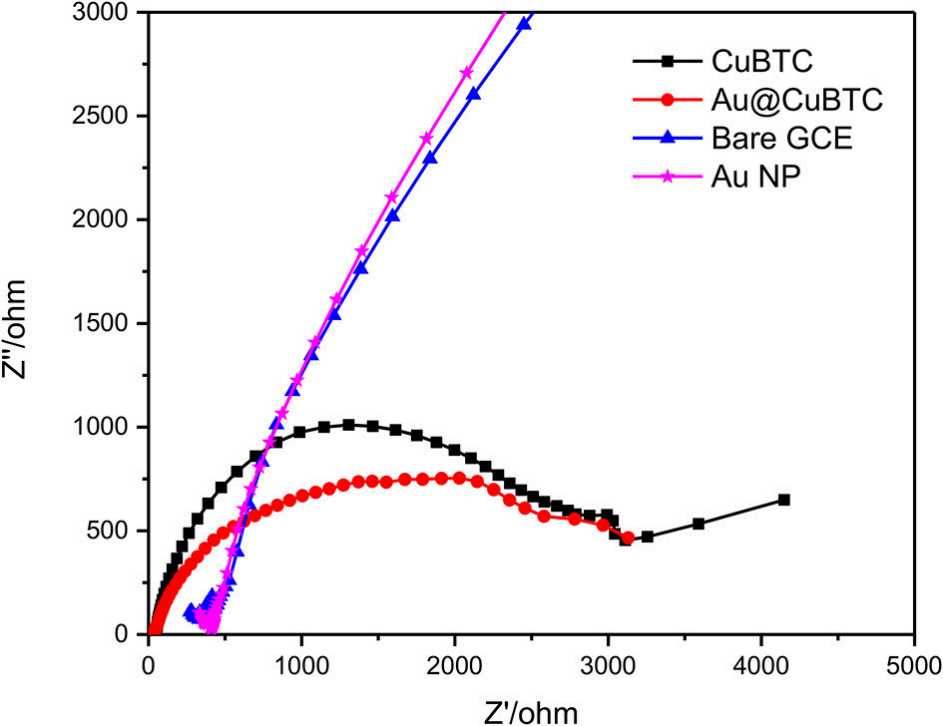
Electrochemical Impedance Spectroscopy (EIS) Nyquist plot of bare GCE, Au NP, CuBTC MOF, and Au@CuBTC MOF.

Bare GCE, Au Np, CuBTC, and Au@CuBTC were characterized by EIS, which is an effective tool to study electrochemical activity by applying variable AC frequencies. The EIS spectra of bare GCE, Au Np, CuBTC, and Au@CuBTC coated films on GCE were recorded in the AC frequency range of 0.01 Hz to 0.1 MHz in a 10 mM [Fe(CN)_6_]^3−/4−^ solution containing 0.1 M KCl. Nyquist plot (Figure 9) showed a slight decrease in a semicircle of Au@CuBTC as compared to CuBTC, which is due to a slight increase in the ionic conductivity in CuBTC after the incorporation of Au nanoparticles due to the small concentration of Au NPs, and a slight difference was observed. Bare GCE and Au Np showed a very small semicircle due to the very low electron transfer resistance (Ret) of the redox probe. The increase in diameter of the semicircle in the CuBTC MOF and Au@CuBTC MOF modified GCE surface was due to the insulating nature of CuBTC MOF which acts as a barrier for the electrochemical process and slowed down the diffusion of the electrochemical probe toward the electrode surface. EIS and CV results are complementary to each other.

## Sensor Performance

**Figure 10:**
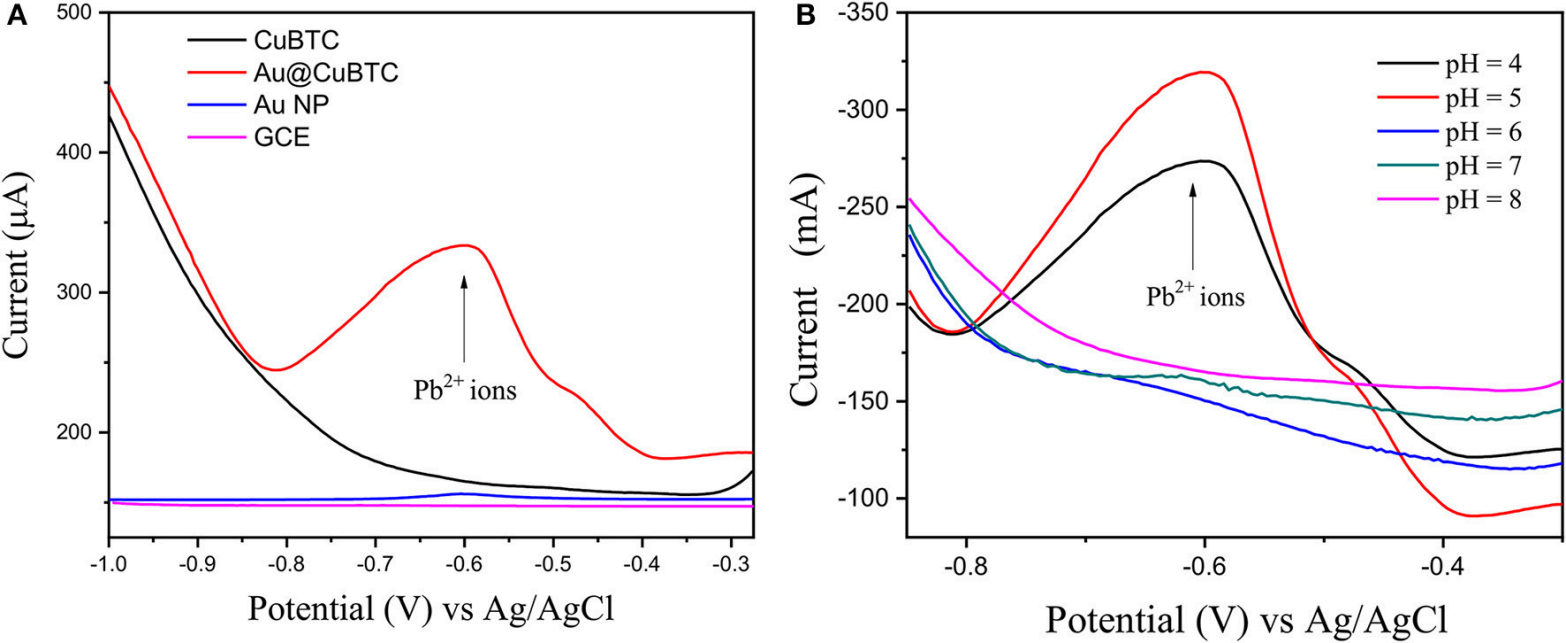
**(A)** Differential pulse voltammograms (DPV) of 0.01 mMol L^−1^ Pb (II) ions in acetate buffer (pH-5) of bare GCE, Au Nps, CuBTC, and Au@CuBTC and **(B)** DPV of Au@CuBTC to 1 μM/L Pb^2+^ ions at various pH.

**Figure 11:**
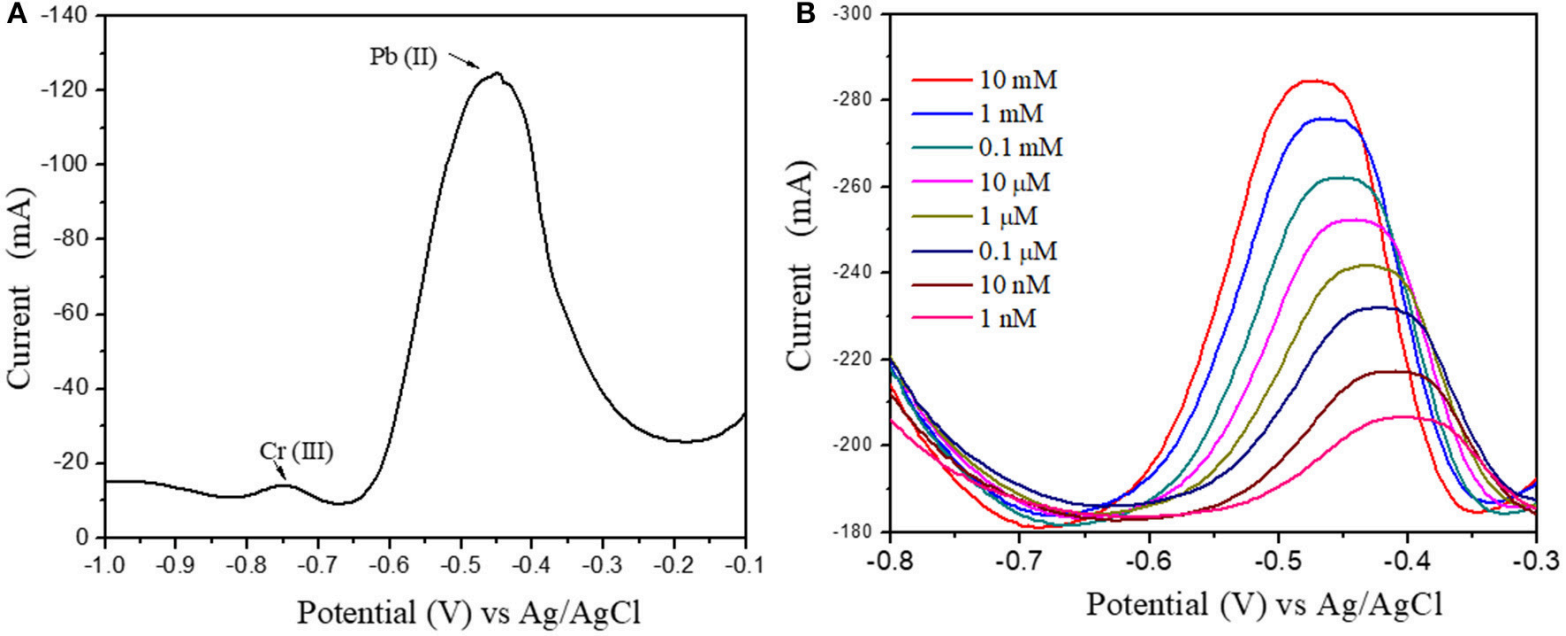
**(A)** Pb(II) and Cr(III) ions DPV response at 1 μM concentration **(B)** Differential Pulse Voltammograms of Au@CuBTC modified GCE electrodes for Pb(II) ions concentration of 10 mM/L to 1 nM/L.

The sensing experiment was carried out in two steps: (1) Accumulation in Pb^2+^ ions solution, and (2) reduction in buffer solution by applying differential pulse voltammetry (DPV), where the accumulated Pb^2+^ ions can get reduced at particular potential Pb^2+^ ions (Equation 1). As shown in Figure 10A, the bare GCE Au nanoparticles coated GCE and CuBTC shows lower response to the Pb^2+^ ions at a concentration of 0.01 mM/L. However, Au@CuBTC shows a significant sensing response to the Pb^2+^ ions, which means that after incorporating Au nanoparticles in CuBTC become sensitive toward Pb^2+^ ions. The Au Np work as a catalyst while the CuBTC MOF provides more surface area and the pore provides more active sites for electrocatalytic activity, which enables highly sensitive detection of Pb^2+^ ions. Here, in the accumulation step, Pb^2+^ ions coordinate with Au@CuBTC MOF and form PbO that are trapped on the surface of Au@CuBTC MOF, while in the DPV they are reduced at the reduction potential (Equation 1) (Metzger, 2012), which results in a large flow of current through working and counter electrodes as shown in Figures 10, 11.

(1) $$\begin{array}{l} {\text{PbO}}_{\left(\text{s}\right)}+{\text{H}}_{2}\text{O}\left(\text{l}\right)+2{\text{e}}^{-1}\to {\text{Pb}}_{\left(\text{s}\right)}+2{OH}_{\left(\text{aq}\right)}^{-1}\\ {\text{E}}^{\circ }=-0.58V \end{array}$$

**Figure 12:**
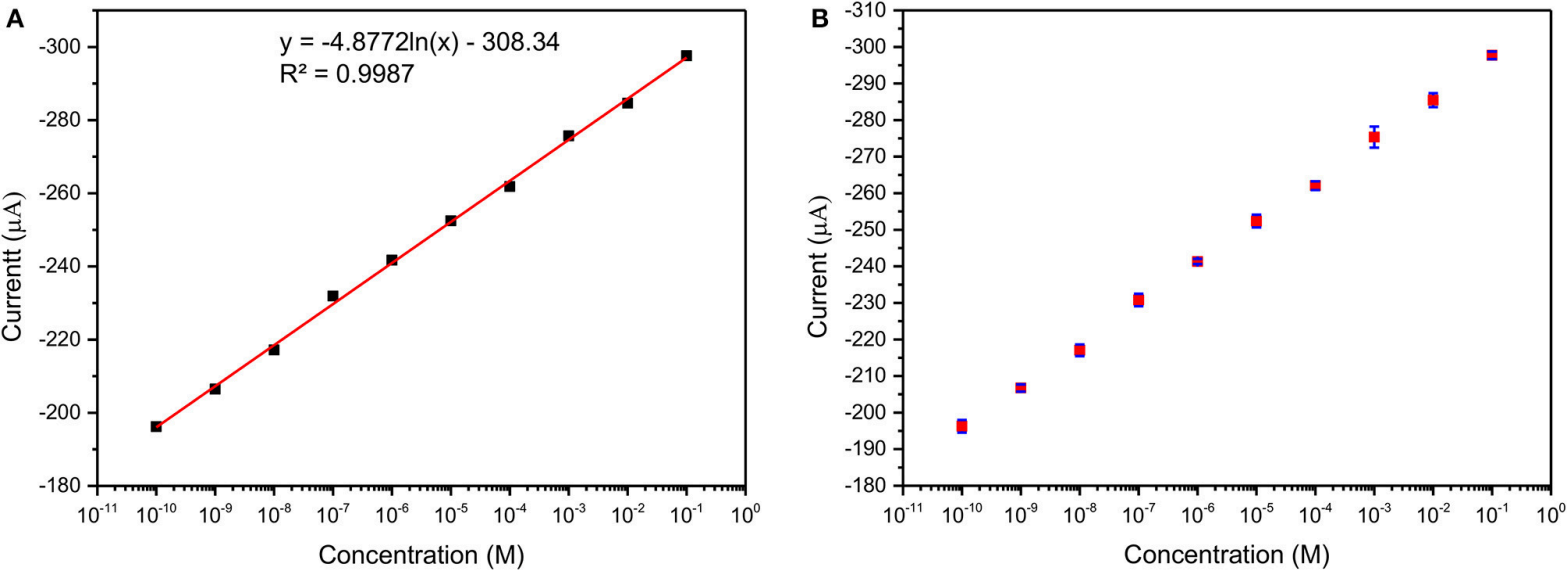
**(A)** Calibration plot and **(B)** Error bar diagram of Au@CuBTC modified GCE electrodes for Pb(II) ions concentration of 10 mM/L to 1 nM/L.

Figure 10B shows the DPV curve of Pb^2+^ ions at the concentration of 1 μM/L at the various pH (4–8) and shows the highest sensitivity at pH 5. All the sensing experiments were carried out in acetate buffer at pH 5. The Au@CuBTC modified GCE electrode was tested for Cr^3+^ ions and Pb^2+^ ions and shows a more selective response toward Pb^2+^ ions as shown in Figure 11A. It was also tested for various concentrations of Pb^2+^ ions in water, as shown in Figure 11. The Au@CuBTC shows a sensing response toward Pb^2+^ ions up to a lower detection limit of 1 nM/L, which is below the Maximum Contamination Level (MCL) of 0.03 μM/L as proposed by EPA, USA (Landmeyer et al., 2003). The comparative studies for the detection of Pb(II) ions by electrochemical method using various materials are shown in Table 3. This indicates that this work has a better deletion limit (LOD) compared to other reported data. The calibration plot and error bar diagram also show (Figures 12A,B) that the fabricated sensor shows good linearity and repeatability.

**Table 3:** Comparative studies for detection of Pb(II) ions by electrochemical method using various materials.

**Sr. No**.	**Sensing material electrode**	**Sensing technique**	**Detection limit (LOD) of Pb^**2+**^ ions**	**References**
1.	Bismuth/Poly(1,8-diaminonaphthalene) modified carbon paste electrode (CPE)	Square-wave voltammetry	0.3 μg L^−1^	Salih et al., 2017
2.	Bismuth-Carbon pate electrode (CPE)	Square-wave anodic stripping voltammetry	0.3 μg/L	Martín-Yerga et al., 2017
3.	Glassy carbon electrode modified SWNTs/Biomass electrode	Differential pulse anodic stripping voltammetry	10^−8^ M	Dali et al., 2018
4.	TAPB-DMTP-COF (TAPB, 1,3,5-tris(4-aminophenyl)benzene; DMTP, 2,5-dimethoxyterephaldehyde; COF, covalent organic framework) modified carbon paste electrode	Differential pulse anodic stripping voltammetry	1.9 nmol/L	Zhang et al., 2018
5.	Iron oxide (Fe_3_O_4_) nanoparticles (NPs) capped with terephthalic acid (TA)/Glassy Carbon Electrode	Square wave anodic stripping voltammetry	0.05 μM/L	Deshmukh et al., 2017
6.	EDTA-Ppy/SWNTs modified stainless steel electrode	Differential pulse voltammetry	0.15 μM/L	Deshmukh et al., 2018a
7.	Fe_3_O_4_@PDA@MnO_2_ electrode	Differential pulse voltammetry	0.03 μg L^−1^	Wang et al., 2020
8.	Au@CuBTC MOF modified GCE electrodes	Differential pulse voltammetry	1 nM/L	This work

## Conclusions

In this study successfully synthesized and rigorously characterized CuBTC MOF and Au nanoparticle incorporated CuBTC MOF (Au@CuBTC). We prepared an electrochemical sensor based on Au@CuBTC MOF that showed a significant response and sensitivity toward Pb^2+^ ions compared with pure CuBTC MOF coated on the GCE electrode. Au@CuBTC MOF shows the highest sensitivity in acetate buffer at pH 5 and shows great affinity toward Pb^2+^ ions in water media at a lower detection limit of 1 nM/L concentration at pH 5.0 by using a DPV technique which is far below the Maximum Contamination Level (MCL) as proposed by EPA, USA.

## Data Availability Statement

The raw data supporting the conclusions of this article will be made available by the authors, without undue reservation.

## Author Contributions

GB and BH contributed equally in experimental work, idea generation, and data analysis. MD and HP contributed for electrochemical experiment data analysis. SMS contributed in experimental work. DP and KP contributed for XPS and XRD experimental data collection analysis. MS contributed as corresponding author (i.e., idea, experiment, data validation, and guidance). All authors contributed to the article and approved the submitted version.

## Conflict of Interest

The authors declare that the research was conducted in the absence of any commercial or financial relationships that could be construed as a potential conflict of interest.
